# Goals-of-care transition after an allogeneic hematopoietic stem-cell transplantation: A cross-sectional survey among hematologists on associated challenges

**DOI:** 10.1038/s41409-026-02873-6

**Published:** 2026-04-27

**Authors:** Anne Pralong, Steffen T. Simon, Marco Herling, Alinda Reimer, Berenike Schoerger, Lisa Pusch, Christoph Scheid, Michael Hallek, Jithmi Weliwitage, Martin Hellmich, Raymond Voltz, Udo Holtick

**Affiliations:** 1 University of Cologne, Faculty of Medicine and University Hospital, Department of Palliative Medicine, Cologne, Germany; 2 University of Cologne, Faculty of Medicine and University Hospital, Centre for Integrated Oncology Aachen-Bonn-Cologne-Duesseldorf (CIO ABCD), Cologne, Germany; 3 University of Cologne, Faculty of Medicine and University Hospital, Centre for Health Services Research (ZVFK), Cologne, Germany; 4 University of Cologne, Faculty of Medicine and University Hospital, Department I of Internal Medicine, Cologne, Germany; 5 Department of Hematology, Cellular Therapy, Hemostaseology, and Infectious Diseases, University of Leipzig, Leipzig, Germany; 6 Comprehensive Cancer Center Central Germany (CCCG) Leipzig-Jena, Leipzig, Germany; 7 University of Cologne, Faculty of Medicine and University Hospital Cologne, Institute of Medical Statistics and Computational Biology (IMSB), Cologne, Germany; 8 University Medical Center Göttingen, Department of Medical Statistics, Göttingen, Germany

**Keywords:** Quality of life, Translational research

To the editor

Patients with hematological malignancies undergoing allogeneic hematopoietic stem-cell transplantation (allo-HSCT) face a (procedural) mortality of about 50% against the background of a real chance of cure [1]. Therefore, timely attention to end-of-life issues during the transplant trajectory is essential in cases of an unfavorable disease course, to prevent detrimental aggressive therapies at the end of life [2, 3]. Yet, compared to patients with solid tumors, those with hematological malignancies near death receive more invasive treatments and have less access to palliative care, which is especially pronounced among allo-HSCT recipients [4]. The time until death after a goals-of-care transition from life-prolonging to end-of-life care (hereafter shortened to “GoC transition”) is often short [5].

Improving end-of-life outcomes in allo-HSCT recipients requires to better understand GoC-transition processes, which remain underexplored. To this end, we conducted a cross-sectional exploratory survey among hematologists to assess challenges and support needs surrounding GoC decision-making and discussions, and to rate the relevance of red flags as clinical indicators to prompt GoC transitions.

We sent an anonymized online survey (LimeSurvey®) to five German university hospitals affiliated with Comprehensive Cancer Centers. Participants had to be hematologists treating allo-HSCT recipients, in the settings of HSCT, general hematology or ICU. We collected data from January to February 2023.

Based on a literature review [4] and on qualitative results within a larger research project [6], we self-developed and pre-tested questions assessing (Supplement 1): challenges related to the timing of GoC transitions and to GoC discussions, ideal timing for GoC discussions versus actual timing in practice, and support needs (multiple choice), as well as perceived relevance of seven red flags, as clinical indicators to prompt GoC transitions (5-point Likert-scale). Additionally, we assessed attitudes toward death (subscale “death-acceptance” of the *Life Attitude Profile-Revised* (LAP-R); subscales “fear of death” and “death avoidance” of the *Death Attitude Profile-Revised* (DAP-R)).

In addition to descriptive statistics, we assessed associations with Pearson chi-square or Fisher’s exact test, Mann-Whitney *U*-test and Spearman’s rho correlation. Distributions of paired nominal data were compared by McNemar test. We considered a two-sided *p*-value of <0.05 statistically significant. We used SPSS Statistics 28.0.1.0 (IBM, Armonk/NY/USA.). Free-text answers were investigated with content analysis.

Fifty-one hematologists (35.3% females, age: *Mdn* = 34 years, range 25–52) started and completed the survey. Residents (51.0%) and attending hematologists (49.0%) were evenly represented and worked in HSCT setting (47.1%), general hematology (35.3%), or ICU (17.6%). For further participants’ characteristics, see Supplement 2. For confidentiality reasons of the participating centers, the number of survey recipients could not be tracked.

*Challenges in determining the appropriate timing for a GoC transition* and *discussing GoC transitions* are presented in Fig. 1a, b, *support options* in Fig. 1d. We report items endorsed by >50% participants to highlight the most salient answers (see Supplements for frequencies of all items).

**Fig. 1:**
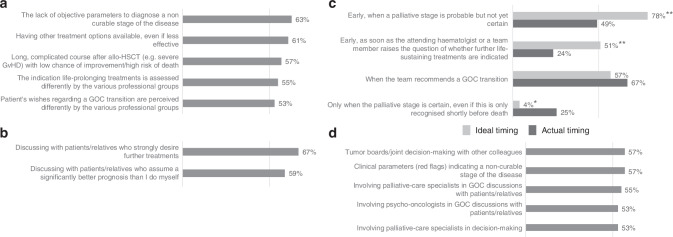
Challenges, support needs and timing of GoC discussions (% hematologists). **a** Challenges selected by >50% of participants in determining the right timing for a GoC transition. **b** Challenges selected by >50% of participants in discussing a GoC transition. **c** Ideal timing for Go**C** discussions versus actual timing in practice (*p < 0.01, **p < 0.001, McNemar Test). **d** Support options wished in the process of GoC transitions and selected by >50% of participants. A full report of all items is provided in the Supplementary Material.

*Ideal timing for GoC discussions versus timing in actual practice* (Fig. 1c): Significant gaps emerged for early time points, considered ideal for GoC discussions but were rarely implemented (*p* < 0.001), and for late timings, where discussions occurred more often than deemed ideal (*p* < 0.01). “When the team recommends a GoC transition” was the most selected timing in actual practice and showed no mismatch with ideal timing (*p* = 0.302). Free-text answers on reasons for gaps revealed hematologists’ death-avoiding attitudes, disagreement within teams on the appropriate timing, communication challenges with patients, or the high prognostic uncertainty.

*Attitudes toward death* were associated with various challenges and support options (Supplement 6). Specifically, “having other treatment options available, even if less effective,” was more frequently perceived as challenging to time GoC transitions, when death acceptance was lower (*p* = 0.026). Similarly, “being confronted with one’s own finitude” more often challenged GoC discussions among hematologists with greater fear of death (*p* = 0.007). Regarding support options, hematologists with lower death acceptance were more likely to involve psycho-oncologists or palliative-care specialists in GoC discussions (*p* = 0.040 and *p* = 0.009, respectively).

*Red flags*: All proposed clinical indicators for GoC transitions were rated as (strongly) relevant. The “combination of severe complications and poorly controlled underlying disease” reached the highest rating (*M* = 4.63, *SD* = 0.57), followed by “Recurrence or progression of the underlying disease without therapy options with proven efficacy/tolerability” (*M* = 4.59, *SD* = 0.71), “Severe, poorly controlled sepsis” (*M* = 4.51, *SD* = 0.68), “Steroid-refractory, severe GvHD that does not respond to second-line therapy” (*M* = 4.43, *SD* = 0.69), “Foreseeable complications of treatment or disease that would no longer allow an autonomous life according to patient’s own statement” (*M* = 4.17, *SD* = 0.69), and “Deterioration of general state with expected prolonged bed rest (ECOG 3/4)” (*M* = 4.06, *SD* = 0.67). “Critical condition with indication for ICU” had the lowest rating (*M* = 3.84, *SD* = 0.97). Free-text responses identified patients’ express wish to limit therapies as an additional red flag.

This exploratory survey first provides comprehensive insights into hematologists’ challenges and needs associated with GoC transitions in allo-HSCT recipients in Germany. Several factors emerged as challenging to define the adequate timing for GoC transitions, which spanned disease and treatment-related issues, and team-related considerations. The absence of objective parameters to diagnose a non-curable stage of the disease was the most frequent challenge, and having such parameters available, one of the most selected support needs. This emphasizes the need for further prognostication research [7]. Our findings on the relevance of specific red flags may help address research gaps in the allo-HSCT-recipients’ population. Further, team discrepancy between professions regarding prognosis or patients’ wishes was another main challenge. This helps raise awareness of team-related barriers to decision-making making and the crucial importance of strengthening team communication.

Challenges posed by GoC discussions were primarily related to patients’ over-expectations regarding prognosis and treatment. The literature highlights the risk of postponing decision-making when facing patients who strive to prolong life [8]. Proficient communication skills are here essential [9]. Notably, when asked about support needs, hematologists strongly preferred involving palliative-care specialists or psycho-oncologists in GoC discussions, rather than participating in communication trainings. This highlights a predilection for on-site, personalized support.

While our survey revealed hematologists’ awareness of the benefits of early GoC discussions, as highlighted in the literature [10, 11], it also identified gaps between ideal and actual timing, underscoring the difficulty of translating ideal considerations into transplant practice. Interestingly, the most frequent selected actual timing for GoC discussions in practice was “when the team recommends a GoC transition”, which showed no mismatch with ideal timing. This highlights how “team intuition” may help supply the lack of objective clinical parameters to early diagnose a palliative stage and timely initiate GoC discussions.

Finally, our findings on attitudes toward death corroborate the possible influence of existential perspectives on decision-making and communication [12]. Confirmation in larger cohorts is warranted.

This survey has several limitations. The small sample size, cross-sectional design, and self-developed items restrict the generalizability of findings. The absence of a calculable response rate also prevents assessment of potential selection bias. Nonetheless, the survey was conducted across multiple leading German centers, which enhances its representativeness. Its methodological development was rigorous.

Our findings underscore the multifactorial challenges associated with GoC transitions in allo-HSCT recipients, while opening research and clinical perspectives to enhance transition processes. This includes promoting prognostication research, addressing team discrepancies while potentializing “team intuition” as a resource, enhancing specific communication skills and raising self-awareness of how one’s own death attitudes may influence practice. Interdisciplinary support by palliative-care specialists and psycho-oncologists may further help optimizing GoC transitions.

## Supplementary information

Supplementary material

## Data Availability

The data that support the findings of this study are available from the corresponding author upon reasonable request.

## Trial registration

This survey is part of the research program “Allo-PaS” (Palliative-Supportive Management in Allogeneic Stem Cell Transplantation), registration number: DRKS00027290, German Clinical Trials Register, https://drks.de/search/en/trial/DRKS00027290.
