# Supplementary material for: Goals-of-care transition after an allogeneic hematopoietic stem-cell transplantation: A cross-sectional survey among hematologists on associated challenges
Source: Bone Marrow Transplant. 2026 Apr 27;61(7):938–40. doi: 10.1038/s41409-026-02873-6 (PMC13349866; doi:10.1038/s41409-026-02873-6)
Supplement: Supplementary file 1 — Supplementary material [file 41409_2026_2873_MOESM1_ESM.docx]

**SUPPLEMENTARY MATERIAL**

*Article title:* Goals-of-care transition after an allogeneic hematopoietic stem-cell transplantation: a cross-sectional survey among hematologists on associated challenges

*Corresponding author:* Anne Pralong, M.D., M.A., University Hospital Cologne, Kerpener Strasse 62, 50924 Cologne, Germany. Email: anne.pralong@uk-koeln.de

**Supplementary material legends**

- Supplement 1: Self-developed survey questions on goals-of-care transitions
- Supplement 2: Hematologists’ characteristics
- Supplement 3: Challenges in determining the adequate timing for a GoC transition (% hematologists, *N* = 51)
- Supplement 4: Challenges in discussing a GoC transition (% hematologists, *N* = 51)
- Supplement 5: Support options wished by participating hematologists (*N* = 51) in the process of GoC transitions
- Supplement 6: Significant associations between challenges/support options, and attitudes towards death (*p*-values)
- Supplement 7: Significant associations between challenges/support options, and demographics (*p*-values)

**Supplement 1: Self-developed survey questions on goals-of-care (GoC) transitions**

| **1. Clinical decision criteria in the process of changing GoC (red flags)** | | | | | | | | | | | | | | | | | | |
| --- | --- | --- | --- | --- | --- | --- | --- | --- | --- | --- | --- | --- | --- | --- | --- | --- | --- | --- |
| ***Here is a list of red flags: How relevant are the following clinical criteria for you to prompt a GoC transition in patients with allo-HSCT?*** | | | | **Not relevant at all** | | **Slightly relevant** | | | **Neither relevant nor irrelevant** | | **Quite Relevant** | | | **Very relevant** | | **Cannot answer** | | |
| - Deterioration of general state of health with expected prolonged bed rest (ECOG 3/4) | | | | 🔿 | | 🔿 | | | 🔿 | | 🔿 | | | 🔿 | | 🔿 | | |
| - Steroid-refractory, high-grade GvHD that does not respond to second-line therapy | | | | 🔿 | | 🔿 | | | 🔿 | | 🔿 | | | 🔿 | | 🔿 | | |
| - Severe, almost uncontrolled sepsis | | | | 🔿 | | 🔿 | | | 🔿 | | 🔿 | | | 🔿 | | 🔿 | | |
| - Recurrence or progression of the underlying disease without therapy options with proven efficacy or tolerability | | | | 🔿 | | 🔿 | | | 🔿 | | 🔿 | | | 🔿 | | 🔿 | | |
| - Foreseeable complications of treatment or disease that would no longer allow an autonomous life (according to patient’s own statement) | | | | 🔿 | | 🔿 | | | 🔿 | | 🔿 | | | 🔿 | | 🔿 | | |
| - Critical condition with indication for ICU | | | | 🔿 | | 🔿 | | | 🔿 | | 🔿 | | | 🔿 | | 🔿 | | |
| - Combination of severe complication and uncontrolled/poorly controlled underlying disease | | | | 🔿 | | 🔿 | | | 🔿 | | 🔿 | | | 🔿 | | 🔿 | | |
| ***Are there any other relevant red flags that have not been mentioned? Please name them briefly. (Free-text answer)*** | | | | | | | | | | | | | | | | | | |
| **2. Challenges in the process of changing GoC (red flags) in patients with allo-HSCT** | | | | | | | | | | | | | | | | | | |
| ***2.1. Here is a list of red flags: How relevant are the following clinical criteria for you to prompt a GoC transition in patients with allo-HSCT?*** | | | | | | | | | | | | | | | | | | |
|  | **0** | **1** | **2** | **3** | **4** | | **5** | **6** | | **7** | | **8** | **9** | | **10** | | **Cannot answer** | |
| 1. How challenging is it for you to determine the timing of a GoC transition, from a medical point of view (medical indication)? | 🔿 | 🔿 | 🔿 | 🔿 | 🔿 | | 🔿 | 🔿 | | 🔿 | | 🔿 | 🔿 | | 🔿 | | 🔿 | |
| 2. How challenging is it for you to discuss a GoC transition with patients/relatives? | 🔿 | 🔿 | 🔿 | 🔿 | 🔿 | | 🔿 | 🔿 | | 🔿 | | 🔿 | 🔿 | | 🔿 | | 🔿 | |
| ***2.2. What makes it challenging for you to determine the timing of a GoC transition for patients with allo-HSCT, from a medical point of view? (multiple choice)*** | | | | | | | | | | | | | | | | | | |
| - The lack of objective parameters for the diagnosis of a non-curable stage of the disease | | | | | | | | | | | | | | | | | | 🔿 |
| - Having other treatment options available, even if they are less effective | | | | | | | | | | | | | | | | | | 🔿 |
| - Disease course with rapid clinical deterioration | | | | | | | | | | | | | | | | | | 🔿 |
| - Long, complicated course after allo-HSCT (e.g. severe GvHD) with very low chance of improvement and high risk of death | | | | | | | | | | | | | | | | | | 🔿 |
| - Deciding on the limitation of certain therapies (e.g. resuscitation, so-called DNR; intensive care, so-called DNI; blood transfusions; anti-infectives; immunosuppressants) | | | | | | | | | | | | | | | | | | 🔿 |
| - Seeing a GoC transition as a failure of my professional duty | | | | | | | | | | | | | | | | | | 🔿 |
| - Situations in which the chance of improvement (and thus the indication life-prolonging treatments) is assessed differently by the various professional groups or disciplines | | | | | | | | | | | | | | | | | | 🔿 |
| - Situations in which the patient's wishes regarding a GoC transition are perceived differently by the various professional groups | | | | | | | | | | | | | | | | | | 🔿 |
| - None of them/Cannot answer | | | | | | | | | | | | | | | | | | 🔿 |
| ***2.3. What is challenging for you when discussing a GoC transition with patients with allo-HSCT and their relatives? (multiple choice)*** | | | | | | | | | | | | | | | | | | |
| - My own fear or reluctance to have such discussions | | | | | | | | | | | | | | | | | | 🔿 |
| - Being confronted with my own finitude | | | | | | | | | | | | | | | | | | 🔿 |
| - Discussing with patients/relatives who assume a significantly better prognosis than I do myself | | | | | | | | | | | | | | | | | | 🔿 |
| - Discussing with patients/relatives who strongly desire further therapies | | | | | | | | | | | | | | | | | | 🔿 |
| - The feeling of not being qualified enough to conduct such discussions | | | | | | | | | | | | | | | | | | 🔿 |
| - Having too little time available for such discussions | | | | | | | | | | | | | | | | | | 🔿 |
| - Being confronted with emotions such as anger, disappointment, fear or sadness | | | | | | | | | | | | | | | | | | 🔿 |
| - None of them/Cannot answer | | | | | | | | | | | | | | | | | | 🔿 |
| **3. Timing of discussions on a GoC transition** | | | | | | | | | | | | | | | | | | |
| ***3.1. When should a GoC transition ideally be discussed with patients with allo-HSCT and their relatives? (multiple choice)*** | | | | | | | | | | | | | | | | | | |
| - Early, when a palliative stage is probable but not yet certain | | | | | | | | | | | | | | | | | | 🔿 |
| - Early, as soon as the attending hematologist or a team member raises the question of whether further life-sustaining treatments are indicated | | | | | | | | | | | | | | | | | | 🔿 |
| - If the team recommends a GoC transition | | | | | | | | | | | | | | | | | | 🔿 |
| - Only when the palliative stage is certain, even if this is only recognised shortly before death | | | | | | | | | | | | | | | | | | 🔿 |
| - None of them/Cannot answer | | | | | | | | | | | | | | | | | | 🔿 |
| ***3.2. When do you actually have such discussions in practice? (multiple choice)*** | | | | | | | | | | | | | | | | | | |
| - Early, when a palliative stage is probable but not yet certain | | | | | | | | | | | | | | | | | | 🔿 |
| - Early, as soon as the attending hematologist or a team member raises the question of whether further life-sustaining treatments are indicated | | | | | | | | | | | | | | | | | | 🔿 |
| - If the team recommends a GoC transition | | | | | | | | | | | | | | | | | | 🔿 |
| - Only when the palliative stage is certain, even if this is only recognised shortly before death | | | | | | | | | | | | | | | | | | 🔿 |
| - None of them/Cannot answer | | | | | | | | | | | | | | | | | | 🔿 |
| ***3.3. If you selected different answers for the previous two questions: What do you think is the reason for that? (free text answer)*** | | | | | | | | | | | | | | | | | | |
| **4. Support in the process of changing GoC** | | | | | | | | | | | | | | | | | | |
| ***Please evaluate the following supportive offers: Which of these are, or would be, helpful for you in making clinical decisions about a GoC transition, as well as for discussing a GoC transition with patients with allo-HSCT and their relatives? (multiple choice)*** | | | | | | | | | | | | | | | | | | |
| - Standardized clinical parameters indicating a non-curable stage of the disease in the clinical course (red flags) | | | | | | | | | | | | | | | | | | 🔿 |
| - HSCT boards and joint decision-making with other colleagues | | | | | | | | | | | | | | | | | | 🔿 |
| - Involving palliative-care specialists in the decision-making process for changing GoC | | | | | | | | | | | | | | | | | | 🔿 |
| - Ethics consultation in case of ethical issues or uncertainty in the process of changing GoC | | | | | | | | | | | | | | | | | | 🔿 |
| - Communication training on breaking bad news | | | | | | | | | | | | | | | | | | 🔿 |
| - Involving psycho-oncologists in GoC discussions with patients/relatives | | | | | | | | | | | | | | | | | | 🔿 |
| - Involving palliative-care specialists in GoC discussions with patients/relatives | | | | | | | | | | | | | | | | | | 🔿 |
| - Observation of GoC discussions by (experienced) colleagues | | | | | | | | | | | | | | | | | | 🔿 |
| - Guidance in GoC discussions with (experienced) colleagues | | | | | | | | | | | | | | | | | | 🔿 |
| - Guidelines (SOP-like) with the most important clinical criteria (red flags) and guidance for GoC discussions | | | | | | | | | | | | | | | | | | 🔿 |
| - None of them/Cannot answer | | | | | | | | | | | | | | | | | | 🔿 |

**Supplement 2: Hematologists’ characteristics**

| **Characteristics (*N* = 51)** | | | **No. (%)** |
| --- | --- | --- | --- |
| Gender (female) | | | *Total* |
| Female |  |  | 18 (35) |
| Male |  |  | 33 (65) |
| Age (years; median and range) | | | 34 (25-52) |
| Medical training | | |  |
| Residents | | | 26 (51) |
| Attending hematologists | | | 25 (49) |
| Health care service | | |  |
| HSCT^1^ | | | 24 (47) |
| General hematology^1,2^ | | | 18 (35) |
| ICU^2^ | | | 9 (18) |
| Experience | | |  |
| Years of clinical practice with allo-HSCT recipients (median and range) | | | 6 (0-25) |
| No. of allo-HSCT recipients per year (median and range) | | | 40 (1-150) |
| No. of patients cared for in the dying phase per year (median and range) | | | 5 (0-40) |
| ^1^ in- and/or outpatients  ^2^ with training in general oncology, hematology, and transplantation. | | | |

**Supplement 3: Challenges in determining the adequate timing for a GoC transition (% hematologists, N = 51)**

**Supplement 4: Challenges in discussing a GoC transition (% hematologists, N = 51)**

**Supplement 5: Support options wished by participating hematologists (*N* = 51) in the process of GoC transitions**

**Supplement 6: Significant associations between challenges/support options, and attitudes towards death (*p*-values)**

| **Attitudes towards death** | **Challenges for adequate GoC-transition timing** | | | **Challenges of GoC discussions** | | | **Support options** | | | | | | | | | | | | | | |
| --- | --- | --- | --- | --- | --- | --- | --- | --- | --- | --- | --- | --- | --- | --- | --- | --- | --- | --- | --- | --- | --- |
|  | Other treatment options available^1^ | | | Own finitude^2^ | | | Psycho-oncologist for GoC discussions^3^ | | | Palliative-care specialists for GoC discussions^4^ | | | Guidance in GoC discussions by colleagues^5^ | | | Observation of GoC discussions by colleagues ^6^ | | | Guidelines/SOP^7^ | | |
| *Mann-Whitney-U test* | *MR S (n)* | *MR NS (n)* | *p* | *MR S (n)* | *MR NS (n)* | *p* | *MR S (n)* | *MR NS (n)* | *p* | *MR S (n)* | *MR NS (n)* | *p* | *MR S (n)* | *MR NS (n)* | *p* | *MR S (n)* | *MR NS (n)* | *p* | *MR S (n)* | *MR NS (n)* | *p* |
| LAP-R (death acceptance) | 22.3 (31) | 31.8 (20) | .026 |  |  |  | 22.0 (27) | 30.5 (24) | .040 | 21.1 (28) | 32.0 (23) | .009 |  |  |  |  |  |  |  |  |  |
| DAP-R: |  |  |  |  |  |  |  |  |  |  |  |  |  |  |  |  |  |  |  |  |  |
| Fear of death |  |  |  | 39.2 (3) | 20.7 (40) | .007 |  |  |  |  |  |  | 16.5 (16) | 25.3 (27) | .026 |  |  |  |  |  |  |
| Death avoidance |  |  |  |  |  |  |  |  |  |  |  |  |  |  |  | 14.7 (15) | 25.9 (28) | .005 | 6.6 (4) | 23.6 (39) | .006 |
| Only items with significant associations are listed. (*P*-values presented are unadjusted. They were no longer significant after adjusting for multiple testing.)  Abbr.: GoC = Goals of Care; MR S = Mean Rank of the group “item Selected”; MR NS = Mean Rank of group “item Not Selected” (A higher “MR S” indicates a higher value of the corresponding Attitudes towards death in the participants’ group who selected the item); n = number of participants in group S or NS; SOP = Standard Operating Procedure.  Full survey item:   1. Having other treatment options available, even if less effective 2. Being confronted with my own finitude 3. Involving psycho-oncologists in GoC discussions with patients/relatives 4. Involving palliative-care specialists in GoC discussions with patients/relatives 5. Guidance in GoC discussions with (experienced) colleagues 6. Observation of GoC discussions by (experienced) colleagues 7. Guidelines (SOP-like) with the most important clinical criteria (red flags) and guidance for GoC discussions | | | | | | | | | | | | | | | | | | | | | |

**Supplement 7: Significant associations between challenges/support options, and demographics (*p*-values)**

| **Demographics** | **Challenges for adequate GoC-transition timing** | | | | | | | | | **Challenges of GoC discussions** | | | **Support options** | | | | | |
| --- | --- | --- | --- | --- | --- | --- | --- | --- | --- | --- | --- | --- | --- | --- | --- | --- | --- | --- |
|  | Long clinical course^1^ | | | Rapid clinical deterioration^2^ | | | Diverging perceptions of patients’ wishes^3^ | | | Feeling under- qualified^4^ | | | Availability of standardized parameters^5^ | | | Tumour boards/ joint decision-making^6^ | | |
| *Mann-Whitney-U test (N = 51)* | *MR S (n)* | *MR NS (n)* | *p* | *MR S (n)* | *MR NS (n)* | *p* | *MR S (n)* | *MR NS (n)* | *p* | *MR S (n)* | *MR NS (n)* | *p* | *MR S (n)* | *MR NS (n)* | *p* | *MR S (n)* | *MR NS (n)* | *p* |
| Age |  |  |  |  |  |  |  |  |  | 12.2 (9) | 29.0 (42) | .001 |  |  |  | 19.5 (22) | 30.9 (29) | .006 |
| Years of clinical practice with allo-HSCT recipients |  |  |  |  |  |  |  |  |  | 12.0 (9) | 29.0 (42) | .001 | 30.4 (29) | 22.2 (22) | .015 | 31.2 (29) | 19.2 (22) | .004 |
| No. of allo-HSCT recipients cared for/year | 30.3 (29) | 20.3 (22) | .016 | 34.1 (16) | 22.3 (35) | .008 |  |  |  |  |  |  |  |  |  |  |  |  |
| No. of patients cared for in the dying phase/year (outliers excluded) |  |  |  |  |  |  | 28.7 (26) | 19.6 (22) | .021 |  |  |  |  |  |  |  |  |  |
| *Fisher exact test (N = 51)* |  |  |  |  |  |  |  |  |  |  |  |  |  |  |  |  |  |  |
| Medical training  (R = Registrar; C = Consultant) |  |  |  |  |  |  |  |  |  | *R:  n (%)* | *C: n (%)* | *p* |  |  |  |  |  |  |
|  |  |  |  |  |  |  |  |  |  | 9 (35%) | 0 (0%) | .002 |  |  |  |  |  |  |
| Only items with significant associations are listed. (*P*-values presented are unadjusted. They were no longer significant after adjusting for multiple testing.)  Abbr.: C = Consultant; MR S = Mean Rank of the group “item Selected”; MR NS = Mean Rank of group “item Not Selected” (A higher MR S indicates a higher value of the corresponding demographics in the participants’ group who selected the corresponding challenge or support option); n = number of participants in group S or NS; R = Registrar.  Full survey item:   1. Long, complicated course after allo-HSCT (e.g. severe GvHD) with very low chance of improvement and high risk of death 2. Disease course with rapid clinical deterioration 3. Situations in which the patient's wishes regarding a GoC transition are perceived differently by the various professional groups 4. The feeling of not being qualified enough to conduct such discussions 5. Standardized clinical parameters that indicate a non-curable stage of the disease in the clinical course (red flags) 6. Tumour boards and joint decision-making with other colleagues | | | | | | | | | | | | | | | | | | |
